# Ecological zonation and phylogeographic structure of *Glossina pallidipes* (Diptera: Glossinidae) in eastern and southern Africa

**DOI:** 10.1016/j.ijppaw.2025.101165

**Published:** 2025-11-22

**Authors:** Attila J. Trájer, Alex Kummer

**Affiliations:** a Sustainability Solutions Research Lab, University of Pannonia, Hungary; b HUN-REN-PE Complex Systems Monitoring Research Group, University of Pannonia, Hungary

**Keywords:** Tsetse fly, African trypanosomiasis, Ensemble models, Phylogeography, One health, Vector ecology

## Abstract

*Glossina pallidipes*, a major vector of African trypanosomiasis, plays a notable role in disease transmission across eastern and southern Africa due to its broad host range, ecological adaptability, and vectorial capacity. This study combined machine learning using 69 environmental, climatic, edaphic, and developmental variables and genetic analysis to delineate the environmental and phylogenetic structuring of *G. pallidipes* populations. Kernel density estimation revealed three primary hotspots in the Ethiopian and East African Highlands and the Northern Zimbabwe Lowveld, with a secondary focus along the Maasai Steppe. Most occurrences were associated with tropical savanna climates and sparsely populated natural and semi-natural landscapes. Ensemble modelling using five algorithms identified temperature seasonality, precipitation of the coldest quarter, and diurnal temperature range as the strongest predictors of occurrence, highlighting the dominant influence of thermal variability over precipitation metrics. Among developmental factors, larviposition and mortality rates during the dry quarter were most influential, reflecting the role of humidity in reproductive success. K-means Clustering revealed three ecologically distinct groups following an east–west gradient, shaped by elevation, aridity, and thermal variability. Decision tree analysis identified the Köppen Aridity Index and elevation as key discriminators among clusters. Phylogenetic analysis of mitochondrial COI sequences demonstrated clear geographic structuring, with a divergent Ethiopian lineage and high connectivity between Kenyan and Tanzanian populations. Frequent co-occurrence with the African buffalo and the common warthog further supports ecological continuity across savanna mosaics. These results reveal that *G. pallidipes* populations are primarily structured by climatic gradients and regional connectivity, informing targeted, region-specific vector control strategies.

## Highlights

• Tropical savanna (Aw) is the dominant climate for *G. pallidipes* occurrence.
• Ensemble models identify precipitation and temperature range as key predictors.
• K-means and decision trees reveal four ecologically distinct tsetse populations.
• Buffalo and warthog most overlap ecologically with *G. pallidipes.*
• Phylogeography shows strong regional structure with an endemic Ethiopian clade.

## Graphical abstract

**Figure:**
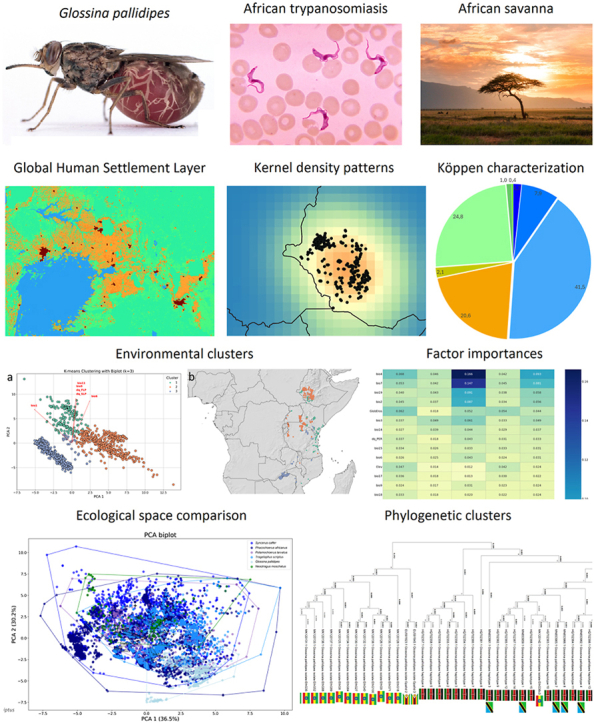

## 1 Introduction

Tsetse flies and trypanosomiasis continue to pose major health and economic challenges across sub-Saharan Africa (Ombura et al., 2024). Among these vectors, *Glossina pallidipes*, Austen 1903, a species of tsetse fly within the family Glossinidae, is epidemiologically relevant as a vector of animal African trypanosomiasis (AAT) (Seyoum et al., 2022) and has been implicated in the transmission of human African trypanosomiasis (HAT) in some endemic areas (Moloo et al., 1992; Vreysen et al., 2013). This viviparous, hematophagous insect is widely distributed across East and Southern Africa (Cecchi et al., 2024), occurring in savannah woodlands and riverine systems (Sciarretta et al., 2010), which provide suitable conditions for survival and reproduction (Ogolla et al., 2023). Current knowledge is largely based either on coarse-resolution distribution maps that provide only a broad picture of the species' range, or on highly localized studies focusing on limited areas. As a result, the environmental thresholds and ecological clusters that define the species’ niche remain poorly resolved.

The broad phenotypic plasticity (Terblanche et al., 2006), wide thermal tolerance window (Terblanche et al., 2008; Basson and Terblanche, 2010), relatively high desiccation tolerance of puparia (Terblanche and Kleynhans, 2009), broad host range (Nyingilili et al., 2016), and high vector competence (Moloo et al., 1992) of *G. pallidipes* enable the maintenance of *Trypanosoma* transmission among wildlife and livestock in certain ecological settings (Malulu et al., 2023). Along with *Glossina morsitans* spp., *G. pallidipes* is the most important species of the subgenus *Glossina* sensu stricto (morsitans) group and one of the major vectors of AAT and HAT in Eastern and Southern Africa (Vreysen et al., 2013).

Unlike many riverine or forest-associated species, *G. pallidipes* often occupies open savannah and woodland mosaics where wildlife, livestock, and human activity intersect (Vreysen et al., 2013; Ogolla et al., 2023). The preferred habitats of this species are open woodlands and bushlands, including Acacia-Commiphora and Acacia-Combretum associations (Bouteille et al., 1998), following the habitat preference and distribution of the mammal hosts (Allsopp et al., 1972; Adabie, 1987). Due to this, *G. pallidipes* is often categorized as a “xeric” (“xerophilic”) species within the morsitans group (Kleynhans and Terblanche, 2011). It should be noted that commonly used ecological descriptors for *Glossina* species, such as “mesophilic” or “xerophilic,” are largely based on the dehydration tolerance of the 3rd and 4th instar larvae (Childs, 2014). Consequently, these terms may not fully capture the ecological breadth or habitat preferences of the species across all life stages.

As was already mentioned, the wide spectra of animal hosts and habitats make *G. pallidipes* an important reservoir of tsetse fly-borne human and animal infections (Bouteille et al., 1998). Increased human-livestock-wildlife interaction facilitates the exchange of trypanosomes between reservoir hosts and humans, complicating disease control efforts (Vicente et al., 2021). However, *G. pallidipes* is weakly anthropophilic, and humans are generally less preferred host for this species. Field studies in Rhodesia showed that *G. pallidipes* and *G. morsitans* Westwood, 1851 responded differently to mobile and stationary baits (Vale, 1974). Stationary baits mainly attracted hungry flies via host odours, aided by visual cues, while mobile baits relied primarily on visual stimuli. Humans were consistently poor stationary baits for *G. pallidipes*, with only extremely hungry flies attempting to feed; nearby domestic animals were preferred by less hungry flies. These findings highlight *G. pallidipes*'s strong avoidance of humans and suggest that feeding and mating behaviours occur in distinct phases, with mobile baits potentially signalling both food and mate opportunities. Another study conducted in interface areas of Kenya indicated that *G. pallidipes* exhibits higher feeding success on wild hosts when available. However, in the absence of preferred wild hosts, these flies often feed on both wild and domestic hosts, including humans (Channumsin et al., 2021). This also suggests that while *G. pallidipes* may prefer wild hosts, it can opportunistically feed on domestic animals and humans when necessary.

From an agricultural perspective, AAT remains one of the most important vector-borne constraints on livestock productivity in Africa (Swallow, 2000; Njiru et al., 2004). Infection with *Trypanosoma congolense* (Broden, 1904), *Trypanosoma vivax* (Ziemann, 1905), and *Trypanosoma brucei* (Plimmer and Bradford, 1899) transmitted by *G. pallidipes* (Moloo et al., 1992), leads to chronic wasting disease, mortality, and loss of agricultural income, with downstream effects on food security and rural livelihoods (Serem, 2024). In endemic areas, regular vector control and chemoprophylaxis are often required to sustain livestock keeping, illustrating the species’ continuing veterinary significance (Swallow, 2000).

The distribution and epidemiological dynamics of *G. pallidipes* are also strongly influenced by environmental and anthropogenic drivers (Kleynhans et al., 2014; Matawa et al., 2020). Land use change, deforestation, climate variability, and agricultural expansion have reconfigured landscapes once inhabited by tsetse, modifying population density, movement patterns, and trypanosomiasis risk (Bourn et al., 2001). In some regions, the habitat destruction has led to localized declines in tsetse populations (Matawa et al., 2020), and community-based tsetse control also resulted in the reduced incidence of trypanosomiasis in local cattle in certain sites (Brightwell et al., 1997). However, the resilience and adaptability of *G. pallidipes* allow it to persist in fragmented habitats and continue serving as a disease reservoir, thereby posing an ongoing threat to both public and veterinary health (Luziga et al., 2017; Bateta et al., 2020). A spatial genetic structuring study across Kenya and northern Tanzania revealed population connectivity despite landscape fragmentation—highlighting the species’ capacity to endure human-modified environments (Bateta et al., 2020).

Control of *G. pallidipes* and the diseases it vectors remains a major challenge for endemic countries (Ombura et al., 2024; Mongare et al., 2025). Past massive control efforts were shown to fail to permanently reduce genetic diversity in *G. pallidipes*, implying recolonization or insufficiently sustained intervention (Okeyo et al., 2017). Traditional control methods include insecticide-treated targets and traps and aerial spraying. Field trials in Zimbabwe found that pyriproxyfen, a juvenile hormone mimic, reduced puparial emergence and larval development in *G. m. morsitans* and *G. pallidipes*. Although some loss of the compound occurred over time, improved persistence could make it a strong candidate for target-based tsetse control (Hargrove and Langley, 1993). For instance, deployment of insecticide-treated targets in the Zambezi Valley (Zimbabwe, 1984–85) caused additional mortality up to 8 % per day in female *G. pallidipes*, leading to local suppression (Hargrove, 2003). A novel dual intervention combining entomopathogenic fungi with SIT (tested in *G. pallidipes*; Ombura et al., 2024) showed strong synergistic suppression, suggesting an innovative biopesticide-SIT integration in vector control (Ombura et al., 2024). Glossinavirus is also a candidate biological agent for tsetse fly control (Kariithi et al., 2013).

In conclusion, *G. pallidipes* plays a notable role in the transmission dynamics of African trypanosomiasis across much of eastern and southern Africa (Makhulu et al., 2020), including the transmission of *Trypanosoma brucei* in certain regions (Bateta et al., 2017). Previous studies have characterized the general distribution and habitat preferences of *G. pallidipes*, highlighting its adaptation to savanna and semi-xeric environments, high desiccation tolerance, and preference for wild ungulate hosts (Terblanche et al., 2008; Channumsin et al., 2021). However, relatively little is known about the spatial co-occurrence of tsetse and their principal hosts, the combined effects of climatic, edaphic, land-cover, and anthropogenic factors, and the environmental thresholds that define ecologically distinct clusters. Integrating occurrence, host distribution, and multidimensional environmental data using modern machine learning and clustering methods can fill these knowledge gaps and support evidence-based interventions. Addressing the epidemiological challenges posed by *G. pallidipes* requires a One Health approach that bridges human health, veterinary care, and environmental management (Mackenzie and Jeggo, 2019), particularly in rural communities where the burden of trypanosomiasis remains high and persistent.

The novelties and scientific contributions of this study are as follows:

1. An integrative analysis of *G. pallidipes* distribution across eastern and southern Africa is presented by combining occurrence records with high-resolution climatic, edaphic, land cover, and host distribution data.
2. Ensemble machine learning methods are used to identify the most influential climatic and ecological factors shaping the occurrence patterns of *G. pallidipes*, with particular emphasis placed on temperature variability, precipitation seasonality, and soil characteristics.
3. Ecologically distinct clusters of *G. pallidipes* are defined based on K-means clustering and decision tree analysis, and clear differentiation between highland and lowland populations is revealed along thermal and humidity gradients.
4. The environmental overlap and spatial co-occurrence between *G. pallidipes* and key mammalian hosts are quantitatively assessed, and the ecological relationships underpinning transmission cycles are highlighted.
5. Ecological clustering is linked with mitochondrial COI phylogenetic structure, and spatially structured lineages of *G. pallidipes* corresponding to major biogeographic regions and ecological gradients are uncovered.

## 2 Materials and methods

### 2.1 Study workflow

The research followed a structured workflow:

1. *Glossina pallidipes* occurrence records were compiled from multiple sources. Duplicate and spatially clustered points were removed using spatial thinning, and all records were resampled to a 2.5 arc-minute grid to harmonize with environmental layers.
2. Occurrence data for five principal wild vertebrate hosts were aggregated into 0.5° grid cells overlapping *G. pallidipes* sites, and the relative frequency of each host within these cells was calculated to assess potential host availability.
3. Climatic, edaphic, land-cover, and human settlement layers were prepared, including temperature-dependent development layers, to provide spatially explicit inputs for predictive models.
4. Köppen-Geiger Climate Classification was applied to understand climatic preferences.
5. Global Human Settlement Layer (GHSL) classification served the purpose of better understanding the habitat range of *G. pallidipes* occurrence.
6. Five machine learning actions (ExtraTrees, Gradient Boosting, Random Forest, Voting Ensemble, XGBoost), were performed to evaluate environmental variable importances.
7. K-means Clustering was used to detect geographical clusters of similar ecological conditions. The identification of environmental zonation can help to develop adequate, region-specific intervention strategies.
8. Principal Component Analysis and convex hull comparisons were conducted to evaluate environmental similarity between *G. pallidipes* and its principal hosts. Geographic overlap of convex hulls was quantified to estimate potential co-occurrence.
9. Decision tree analysis was applied to determine the environmental variables distinguishing the identified clusters. The use of decision tree classifiers allows for a hierarchical understanding of how environmental conditions partition ecologically based population clusters—an approach that highlights nonlinear ecological thresholds that are difficult to capture through linear models.

Supplementary figure S1 visualizes the main workflow steps.

### 2.2 Source of presence-absence data and its processing

#### 2.2.1 *Glossina pallidipes* occurrence data

The geographic distribution of *G. pallidipes*, including both presence and absence sites, was compiled from multiple sources. The core dataset was derived from Cecchi et al. (2024), who synthesized occurrence records from 219 scientific publications covering the period 1990–2020 (Table 25 in Cecchi et al., 2024). To enhance the spatial and temporal resolution of the dataset, national tsetse and trypanosomosis atlases and regionally focused studies were also integrated. These included data from Kenya (Ngari et al., 2020), Ethiopia (Gebre et al., 2022), Zimbabwe (Shereni et al., 2021), and Rwanda (Gashururu et al., 2021), as well as site-specific surveys and modelling studies from Tanzania (Manangwa et al., 2019), Kenya (Okoth et al., 2007; Gachoki et al., 2021), and the continental atlas of *Glossina* distribution and infection (Cecchi et al., 2015). Additional occurrence data was achieved from the Global Biodiversity Information Facility dataset (GBIF, *Glossina pallidipes*, 2025).

Based on the combined sources, *G. pallidipes* occurrences were confirmed in nine African countries: Ethiopia, Kenya, Malawi, Mozambique, Rwanda, Uganda, the United Republic of Tanzania, Zambia, and Zimbabwe.

#### 2.2.2 Principal vertebrate host occurrence data

Occurrence data for five principal wild vertebrate hosts of *G. pallidipes* based on Ogolla et al. (2023)—the African buffalo (*Syncerus caffer* (Sparrman, 1779)), common warthog (*Phacochoerus africanus* (Gmelin, 1788)), harnessed bushbuck (*Tragelaphus scriptus* (Pallas, 1766)), bushpig (*Potamochoerus larvatus* (F. Cuvier, 1822)), and suni (*Neotragus moschatus* (Von Dueben, 1846))—were downloaded from the Global Biodiversity Information Facility (Gbif, 2025, Gbif, 2025, GBIF, Phacochoerus africanus, 2025, GBIF, Syncerus caffer, 2025, GBIF, Tragelaphus scriptus, 2025). For each species, occurrence records were filtered to include only those marked as “present” and were exported in simple tab-separated values (TSV) format.

While naturally many other African mammal species could have been included, these five were selected for their strong indicator value as principal hosts, providing a practical representation of host availability and habitat associations relevant to *G. pallidipes* ecology.

#### 2.2.3 Processing of occurrence data

To harmonize the occurrence and pseudo-absence data of *G. pallidipes* with the spatial resolution of the environmental datasets and mammal records, all coordinates were resampled to a 2.5 arc-minute (∼0.0417°) grid, corresponding to the resolution of WorldClim and other climatic data sources used in the analysis. The input dataset consisted of two-point layers: one containing confirmed presence records and another representing surveyed locations where the species was not detected. Each record was expressed in decimal latitude and longitude format. All coordinates were assigned to their respective grid cells, and the centroids of these cells were used to represent the spatial position of each record. Duplicate cells were removed to retain a single entry per grid. Absence cells overlapping with presence cells were excluded to ensure mutually exclusive datasets. The resulting grid-based presence and absence datasets were exported into separate sheets of an Excel file for subsequent analysis.

Fig. 1 shows the presence and absence points related to *G. pallidipes*.

**Fig. 1:**
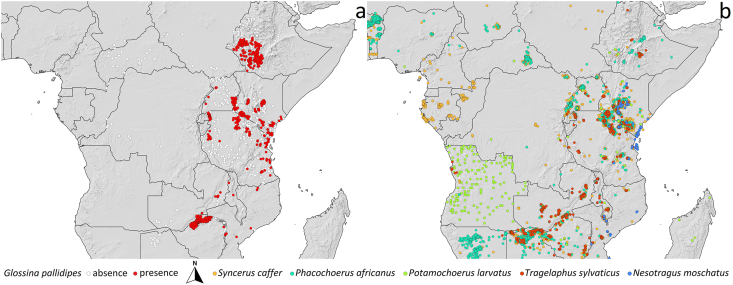
Presence and absence sites of *Glossina pallidipes* (a) in relation to five key African wild mammal taxa that serve as common blood-feeding hosts (b).

To facilitate spatial comparison, all occurrence points were aggregated into 0.5° grid cells across Africa. The percentage occurrence of each mammal species within the *G. pallidipes* grid cells was then calculated, and the relative proportions were visualized using pie charts.

To assess the environmental similarity between *G. pallidipes* and its principal mammalian hosts, a principal component analysis (PCA) was conducted based on 19 bioclimatic variables (WorldClim v2.1), the Köppen aridity index, elevation above sea level, soil type, global human settlement level (GHSL), Köppen climate classification, and multiple ecological parameters associated with *G. pallidipes* developmental requirements.

The convex hull centroids of each species in the PCA space were subsequently compared using hierarchical cluster analysis (HCA). The relative overlap between the convex hull of *G. pallidipes* and those of the five mammal species was then quantified to determine which host exhibited the greatest environmental overlap within the fly's distributional range.

### 2.3 Environmental factors used

To determine the degree of urbanization at positive and negative trapping sites, we accessed Global Human Settlement Layer data (GHS-SMOD; Pesaresi et al., 2024) provided by EC JRC. Elevation data were obtained from the ETOPO Global Relief Model (MacFerrin et al., 2024).

Global rainfall erosivity data were sourced from the European Soil Data Centre (ESDAC) of the European Commission, based on the work of Panagos et al. (2022). To extract data for all trapping sites, raster values were sampled with QGIS 3.34.1.

Climate zones within the study area were categorized using the Köppen-Geiger classification map at 1-km resolution, developed by Beck et al. (2018), based on the WorldClim version 2.1 dataset (Fick and Hijmans, 2017). This classification scheme provides a categorical representation of prevailing climatic conditions.

To characterize the climatic conditions associated with *G. pallidipes* occurrences, 19 standard bioclimatic variables were extracted from WorldClim version 2.1 (Fick and Hijmans, 2017). These variables represent long-term averages for the period 1970–2000 and include key metrics related to temperature and precipitation seasonality and extremes.

### 2.4 Kernel density estimation (KDE)

Kernel Density Estimation (KDE) is a widely applied technique in biostatistics and epidemiology for identifying spatial hotspots (Chen, 2017). In this study, KDE was used to visualize the spatial distribution of *G. pallidipes*. A Gaussian kernel with a bandwidth of 1.0° was applied, and the output was generated at a spatial resolution of 0.25° (approximately 25–30 km). The resulting density surfaces were converted to raster format and visualized using QGIS 3.34.11 in combination with GRASS GIS 8.4.0 software.

### 2.5 K-means clustering

K-means Clustering was applied to group occurrence sites into distinct environmental clusters (Ikotun et al., 2023). The algorithm iteratively assigned data points to the nearest centroid across k clusters, minimizing intra-cluster variation. The optimal k was determined using the Elbow Method (based on Within-Cluster Sum of Squares) and Silhouette Score, which respectively measure compactness and separation (Saraswat et al., 2023). Clustering results were visualized to identify underlying ecological groupings.

### 2.6 Estimation of temperature-dependent *Glossina* development and mortality rates

To estimate *Glossina* developmental dynamics under dry- and wet-quarter thermal conditions, temperature-dependent life history parameters were calculated using the bioclimatic variables bio8 (mean temperature of wettest quarter) and bio9 (mean temperature of driest quarter) derived from the WorldClim dataset in QGIS. These raster-based temperature layers were used as spatial predictors to compute adult and pupal mortality, pupal emergence, and larviposition rates according to empirically fitted equations based on previously published laboratory and field studies (Lord et al., 2018).

Fitted thermal response equations.

Four temperature-dependent functions were fitted to experimental and field-derived data describing *G. pallidipes* survival and reproduction:

i) Adult female mortality rate per day (Eq. (1)).

Estimated from mark–recapture experiments on Antelope Island, Zimbabwe (Hargrove, 2004):

$$AFM=0.135+\frac{0.0254-0.135}{1+{\left(\frac{T_{q}/10}{32}\right)}^{13.86}}\;if\;10\leq T_{q}\leq 35;\;T_{q}<10\;\to \;AFM\;at\;T_{q}=10;\;T_{q}\;>\;35\;set\;to\;AFM\;at\;T_{q}=35$$

where *AFM* is the adult female mortality rate per day, *T*_*q*_ is the mean daily temperature of a quarter of the year.

ii) Pupal mortality rate per day (Eq. (2)):

Derived from laboratory experiments (Phelps, 1973):

$$PMR=0.199-0.0184T_{q}+0.000501{T_{q}}^{2}-0.00000332{T_{q}}^{3}\;if\;10\leq T_{q}\leq 35$$

where *PMR* is the pupal mortality rate per day, *T*_*q*_ is the mean daily temperature of a quarter of the year.

iii) Pupal emergence rate per day (Eq. (3)):

Based on laboratory emergence data fitted following Phelps and Burrows (1969):

$$PER=0.0593-0.00976T_{q}+0.000548{T_{q}}^{2}-0.00000783{T_{q}}^{3}\;if\;10\leq T_{q}\leq 35$$

where *PER* is the pupal emergence rate per day, and *T*_*q*_ is the mean daily temperature of a quarter of the year.

iv) Larviposition rate per day (Eq. (4)):

Derived from published field observations (Hargrove and Langley, 1993; Hargrove, 2001), with separate fits for first and subsequent larviposition events.

First larviposition:

$$FLP=0.00195T_{q}+0.0145\;if\;15\leq T_{q}\leq 35$$

where *FLP* is the first larviposition rate per day, and *T*_*q*_ is the mean daily temperature of a quarter of the year.

Subsequent larviposition:

$$SLP=0.00538T_{q}-0.0213\;if\;15\leq T_{q}\leq 35$$

where *SLP* is the subsequent larviposition rate per day, and *T*_*q*_ is the mean daily temperature of a quarter of the year.

Each equation was implemented in the QGIS raster calculator to derive spatially explicit surfaces of temperature-dependent rates, using bio8 and bio9 as input variables representing wet- and dry-quarter mean temperatures, respectively. The resulting maps quantify geographic variations in adult and pupal mortality, pupal emergence, and larviposition potential under the respective seasonal thermal regimes. These outputs were subsequently used as *Glossina*-development-based environmental layers in the ensemble modelling framework described above.

Finally, the analyses incorporated a total of 69 variables, categorized as follows: 20 climatic variables (19 bioclimatic variables plus the Köppen Aridity Index), 21 edaphic (soil) factors (FAO soil orders) occurring within the study region, 9 climatic classes (Köppen climatic classification), 2 topography-related variables (elevation and rainfall erosivity index), 7 urbanization variables (Global Human Settlement Layer settlement classes), and 10 *Glossina* development factors.

Supplementary table S1 shows the applied environmental and *Glossina* development factors.

### 2.7 Machine learning classification models

To understand the background of the presence-absence status of *G. pallidipes*, supervised machine learning framework was applied integrating multiple ensemble classification algorithms. This approach allowed for robust prediction and interpretation of environmental and ecological drivers of species occurrence, based on both continuous and categorical variables.

#### 2.7.1 Data preparation and preprocessing

The input dataset was prepared in tabular format and included the binary response variable ("Status") along with multiple predictor variables describing climatic, edaphic, and land-cover conditions. Among the predictors, three categorical variables—Köppen climate classification, soil type, and GHS-SMOD class—required transformation prior to analysis. These categorical features were encoded using one-hot encoding (Rodríguez et al., 2018) via a ColumnTransformer (Galli, 2020), with the first level of each categorical variable dropped to prevent redundancy and multicollinearity (i.e., the "dummy variable trap"). The resulting feature matrix contained both the binary-encoded categorical variables and the continuous predictors in a unified structure, suitable for tree-based algorithms.

The cleaned and encoded dataset was then partitioned into training and testing subsets using an 80/20 split, with a fixed random seed (random_state = 42) to ensure reproducibility. The training data were used to fit the models, while the test data served to evaluate predictive performance.

#### 2.7.2 Ensemble classification models

Four ensemble classification algorithms were employed using their default or commonly accepted hyperparameter settings:

• Random Forest (RF): A bagging ensemble of decision trees trained on bootstrap samples (Pal, 2005), using random feature subsets at each split (n = 100 estimators).
• Gradient Boosting Classifier (GB): A boosting-based ensemble that sequentially fits new trees to the residuals of previous trees to minimize a loss function (Natekin and Knoll, 2013).
• Extra Trees Classifier (ETC): A randomized tree ensemble similar to Random Forest but introducing additional randomness by selecting split thresholds at random (Sharaff and Gupta, 2019).
• Extreme Gradient Boosting (XGBoost): An optimized gradient boosting implementation that uses regularized learning to prevent overfitting (Chen and He, 2015). It was configured with eval_metric = 'logloss' and use_label_encoder = False to ensure compatibility with the scikit-learn pipeline and suppress deprecation warnings.
• In addition to the individual classifiers, a soft-voting ensemble classifier was constructed using the VotingClassifier in scikit-learn (Bandi et al., 2023).

#### 2.7.3 Model training and evaluation

All models were trained on the training subset and evaluated on the test subset using two widely accepted classification metrics: accuracy (the proportion of correct predictions) and weighted F1-score, which accounts for both precision and recall while adjusting for class imbalance.

Each classifier's performance was reported to enable model comparison. The use of multiple models also allowed for the quantification of feature importance variability, providing more robust ecological inference regarding the drivers of *G. pallidipes* distribution.

#### 2.7.4 Feature importance analysis

To assess the influence of individual predictors, feature importance scores were extracted from all classifiers that support them natively (i.e., all except classifiers without the feature_importances_ attribute). For the soft-voting ensemble, feature importance was computed as the mean of the importance scores from its constituent base models. This strategy mitigates model-specific biases and highlights consistently informative predictors (Sagi and Rokach, 2018). All feature importance scores were compiled into a single data matrix. Feature names were cleaned to remove technical prefixes introduced by the encoding process, ensuring readability in the final outputs.

For the predictor set used in the *G. pallidipes* modelling framework, the correlation structure among variables was assessed using pairwise correlation matrices (Fig. S2–S6). The identification of strong inter-variable correlations was intended to reduce redundancy in the dataset, thereby limiting potential performance loss and enhancing the model's ability to generalize to previously unsampled environments.

### 2.8 Decision tree analysis

A decision tree classifier (Kushwah et al., 2022) was used to interpret the K-means Clustering results. Environmental and categorical variables (e.g., climate, land cover, soil) were pre-processed, and clusters were used as target classes. The tree revealed which variables most effectively distinguished the clusters, providing an interpretable model of cluster membership. This approach bridges unsupervised structure detection with supervised rule-based interpretation, offering insights into the environmental drivers of *G. pallidipes* occurrence.

### 2.9 Performance of phylogenetic time tree analysis

Mitochondrial cytochrome *c* oxidase subunit I (COI) gene sequences of *G. pallidipes* were obtained from the National Center for Biotechnology Information (NCBI) GenBank database (Wheeler et al., 2000). The published genetic information was based on the articles of Petersen et al. (2007), Dyer et al. (2008), Ouma et al. (2011), Rodrigues et al. (2019), and Bateta et al. (2020).

All sequences were aligned using the ClustalW algorithm (Hung and Weng, 2016) implemented in MEGA 11 (Tamura et al., 2021), applying default alignment parameters. The multiple sequence alignment was manually inspected and trimmed to ensure proper homology and to remove ambiguous regions.

Phylogenetic relationships among the COI haplotypes were inferred using the neighbour-joining (NJ) method (Saitou and Nei, 1987) in MEGA 11. The evolutionary distances were computed using the p-distance model (Strimmer et al., 2003), which calculates the proportion (p) of nucleotide sites at which two sequences differ, without correction for multiple substitutions. To assess the robustness of the inferred tree topology, a bootstrap analysis with 1000 replicates was conducted. All positions containing gaps and missing data were eliminated (complete deletion option), and the final dataset included only aligned positions shared across all sequences. Glossina_pallidipes_COI_FASTA.txt file contains the used nucleic acid data.

## 3 Results

### 3.1 Density patterns

Kernel density analysis indicates that the occurrence sites of *G. pallidipes* are primarily concentrated in three major regions of eastern and southern Africa: the Ethiopian Highlands; the East African Highlands, particularly in central and western Kenya and northern Tanzania, including the Serengeti Plain; and the Northern Zimbabwe Lowveld, together with adjacent low-to mid-altitude areas of Zambia and Mozambique. These regions exhibit the highest relative density values, shown in red on the kernel density map, representing the core distribution zones with numerous occurrence records. A secondary hotspot is evident along the Maasai Steppe, extending from northeastern Tanzania toward southern Kenya. This area likely functions as a transitional zone between highland and lowland populations, potentially facilitating dispersal or persistence in fragmented habitats (Fig. 2).

**Fig. 2:**
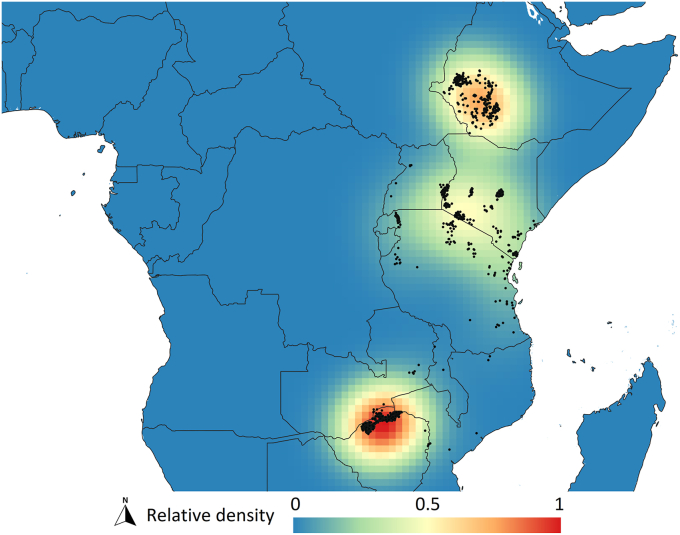
Kernel density patterns of *Glossina pallidipes* occurrence.

### 3.2 Habitat characterization

#### 3.2.1 Climatic conditions

The majority of *G. pallidipes* occurrence sites (approximately 51.5 %) are in tropical climate zones, as defined by the Köppen-Geiger classification system (Af, Am, Aw). The dominant climate type is tropical savanna (Aw), which alone accounts for nearly 41.5 % of the recorded sites. This climate zone is characterized by pronounced wet and dry seasons, supporting woodland, bushland, and grassland mosaic environments well-suited to the ecological needs of *G. pallidipes*, including host availability, suitable vegetation for resting, and access to humid microhabitats that prevent desiccation, such as shaded areas and moist soil for pupal development.

Substantial portions of the sites also occur within semi-arid (BSh, ∼20.6 %) and subtropical highland climates (Cwa, Cwb, and Cfb, ∼26.2 %). These regions, especially in East and Southern Africa, include elevated plateaus and highlands, such as the Kenyan Highlands, Tanzanian uplands, and the Zimbabwean Highveld. Such areas offer moderated temperatures and vegetation cover, which may buffer tsetse populations from climatic extremes and provide persistent habitats during seasonal transitions.

The presence of *G. pallidipes* in Af (tropical rainforest) and Am (monsoon) zones (∼1.7 % and ∼7.9 % respectively) is less frequent but notable, possibly representing populations near dense forest edges or riverine systems. Only a very small proportion of records fall within Mediterranean-type (Csb) or cool temperate (Cfb) regions, reflecting ecological limits of the species in terms of temperature and humidity tolerances.

Spatial overlay also shows clear geographic clustering of *G. pallidipes* records within Aw-dominated belts in Kenya, Tanzania, and Zambia, as well as significant presence along climate transition zones between tropical and subtropical classes. These patterns support the conclusion that *G. pallidipes* favors intermediate climatic conditions with seasonal variability and moderate water stress, avoiding both extreme aridity and dense evergreen forests (Fig. 3a and b).

**Fig. 3:**
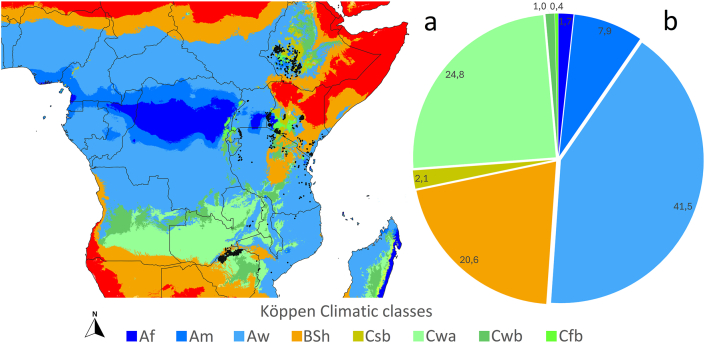
The occurrence of *Glossina pallidipes* projected to the Köppen-Geiger climatic classification map (a) and the pie chart representation of the occurrences of these sites by Köppen climatic classes (b).

#### 3.2.2 Urbanization level

The majority of *G. pallidipes* occurrence sites are associated with sparsely populated rural landscapes. Specifically, nearly 71 % of sites fall within very low-density rural areas, while an additional 18.6 % are in low-density rural regions. Together, these two categories account for approximately 89.6 % of all recorded sites. Only a small fraction of sites (0.8 %) are found within rural clusters, which represent slightly more aggregated human settlements, and suburban or peri-urban areas account for just under 10 % of site occurrences (9.1 %). This suggests that *G. pallidipes* tends to avoid denser human settlements and urban peripheries, likely due to habitat modification, reduced vegetation cover, and vector control activities in more developed areas (Fig. 4).

**Fig. 4:**
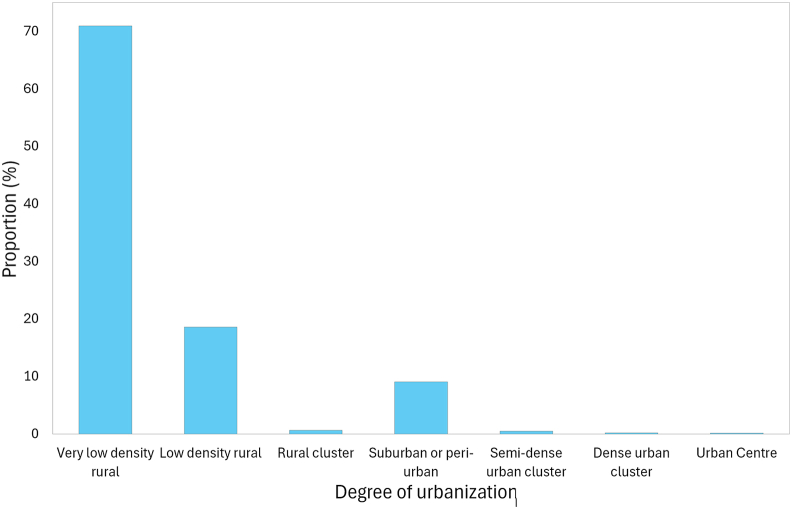
The occurrences of the sites by GHSL land cover categories.

### 3.3 Ensemble results

#### 3.3.1 Evaluation of ensemble feature importance

Based on the feature importance heatmap derived from the ensemble classification models, a clear shift emerged in the relative influence of environmental predictors associated with the presence of *G. pallidipes*. Among the evaluated variables, temperature-related predictors—particularly bio4 (temperature seasonality) and bio7 (temperature annual range)—consistently exhibited the highest relative importance across the ensemble models. The Gradient Boosting and XGBoost classifiers highlighted these two variables as dominant contributors, with bio19 (precipitation of the coldest quarter) and bio2 (mean diurnal temperature range) following as secondary influences. The Random Forest and Voting Ensemble algorithms presented a more balanced distribution of importance among climatic and environmental variables, including moderate contributions from GlobEros and Elevation. Collectively, the results emphasize the relatively strong role of temperature variability and seasonality over precipitation metrics in shaping the predicted distribution of *G. pallidipes* (Fig. 5). Supplementary Table S2 shows the accuracy and F1-score results of ensemble methods.

**Fig. 5:**
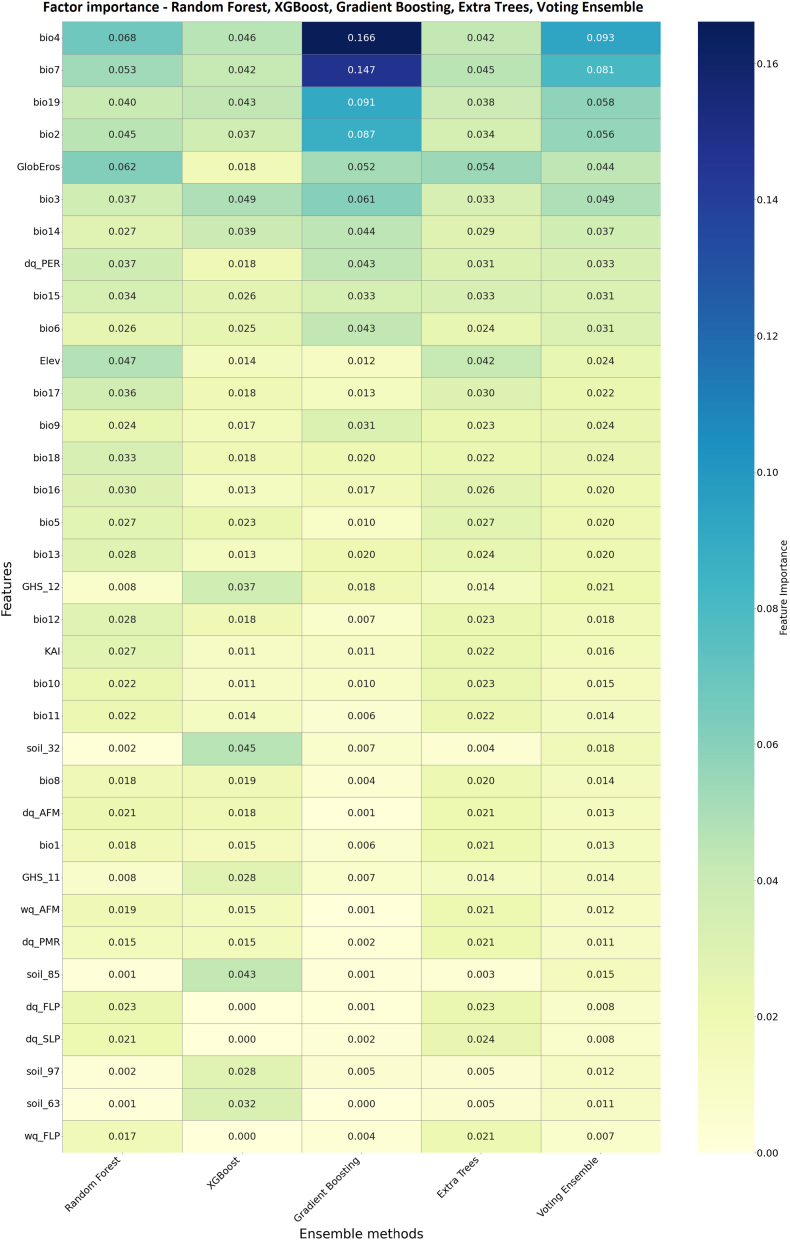
Importance of environmental and *Glossina* development factors, estimated using an ensemble of five factor importance analysis models. Only the top 35 variables were displayed.

The accuracy values of the ensemble models range between 0.783 and 0.826, the F1-scores are between 0.782 and 0.807. The highest accuracy and F1-score values can be found in the case of the Random Forest and Voting Ensemble methods.

##### 3.3.1.1 Evaluation of ensemble feature importance across variable factor groups

Temperature-related predictors again dominated model performance. Temperature seasonality (bio4) and temperature annual range (bio7) consistently ranked highest across all ensemble algorithms, with the strongest influence detected in the Gradient Boosting and XGBoost models. Precipitation of the coldest quarter (bio19) and mean diurnal temperature range (bio2) contributed to secondary but consistent importance, suggesting that both thermal variability and cold-season precipitation exert key constraints on *G. pallidipes* occurrence (Fig. 6a).

**Fig. 6:**
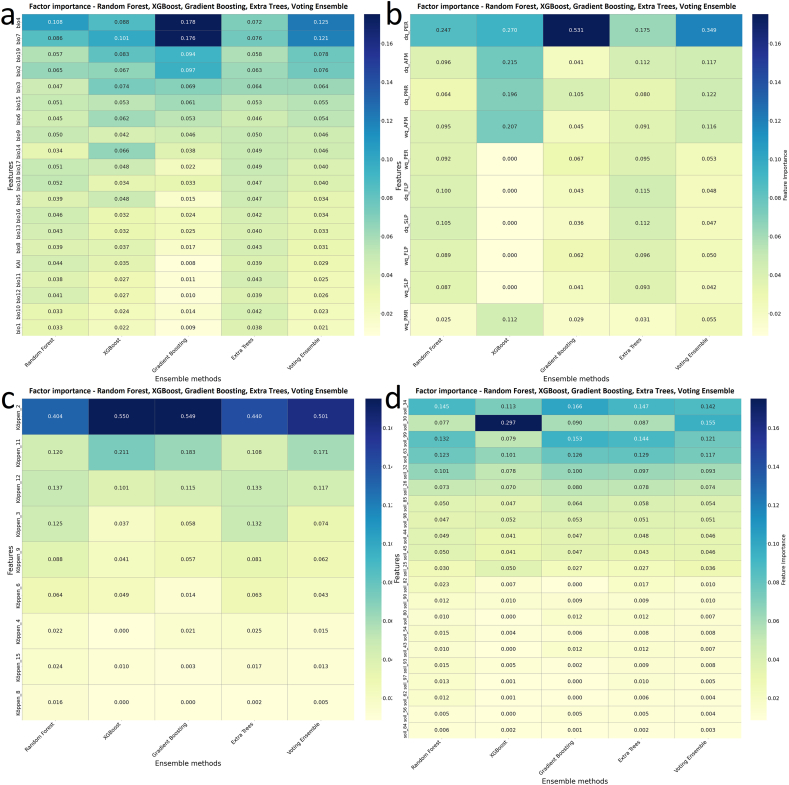
Factor importances of environmental and *Glossina* development variables, assessed using an ensemble of five factor importance methods. Panels show: (a) bioclimatic variables, (b) *Glossina* development variables, (c) Köppen climatic classes, and (d) FAO soil types.

Among the *G. pallidipes* development variables, the ensemble results emphasized the dry quarter's subsequent larviposition rate per day (dp_PER) as the most dominant predictor. This finding highlights the importance of humidity conditions in the reproduction capability of *G. pallidipes* following the first larviposition occasion. Secondary and tertiary contributions from the dry quarter's adult female mortality rate per day (dq_AFM) and pupal mortality rate per day (dq_PMR) also support the ecological linkage between *G. pallides* development and environmental humidity (Fig. 6b).

The models identified strong clustering of importance within a few Köppen categories, with the predominance of tropical, monsoon (Köppen_2, Am) type showing dominant influence across all algorithms. This pattern indicates that *Glossina* occurrence is most strongly associated with tropical, humid environments characterized by moderate temperature seasonality and year-round precipitation. The second most important predictor, the dry winter, hot summer temperate (Köppen_11, Cwa) refers to the occurrence of *G. pallidipes* in humid temperate climate tropical highlands (Fig. 6c).

FAO soil-related predictors showed a more distributed importance pattern, though XGBoost and Gradient Boosting assigned high relevance to soil_32 (vertisols) and soil_85 (fluvisols), followed by soil_63 (nitisols). These results underline the significance of fine-textured and moisture-retaining soils, which are conducive to larval development and pupal survival and positively influence the presence of mammal hosts, e.g., in riparian habitats. Supplementary Table S3 summarizes the accuracy and F1-score values of the ensemble models across the climatic, developmental, Köppen, and edaphic factor groups. These values suggest that climatic factors have the highest predictive values in the determination of *G. pallidipes* occurrences (Fig. 6d).

### 3.4 K-means clustering results

Based on the Elbow and Silhouette methods, the optimal number of ecological clusters for *G. pallidipes* was three (Fig. S7). The PCA biplot shows that the three clusters are well separated along the first two principal components, which together capture the major environmental gradients of the species’ ecological niche. The most important variables contributing to the separation of the clusters were the minimum temperature of the coldest month (bio6), the mean temperature of the driest quarter (bio9), the mean temperature of the coldest quarter (bio11), and the rates of first and subsequent larviposition during the driest quarter (dq_FLP, dq_SLP). These parameters suggest that temperature and reproductive dynamics under arid conditions are key ecological factors differentiating *G. pallidipes* populations (Fig. 7a).

**Fig. 7:**
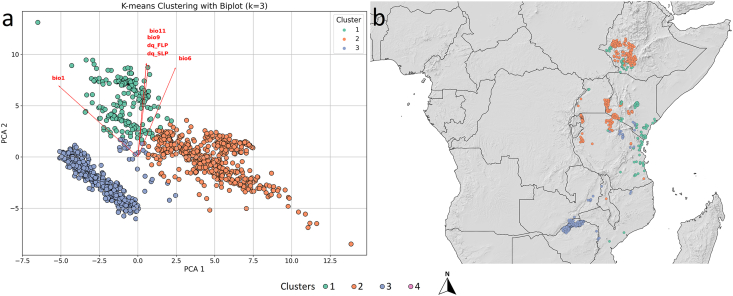
Ecological clusters of *Glossina pallidipes* determined by K-means Clustering (a), and mapped clusters (b).

Cluster 1 occupies the upper region of the ordination space (Fig. 7a) and is characterized by relatively moderate temperature conditions and intermediate values of larviposition rates. Geographically, Cluster 1 sites are mostly located in the Ethiopian Highlands and central parts of East Africa (Fig. 7b). Cluster 2, positioned in the central area of the PCA plot, is primarily defined by high elevation (elev) and increased pupal development rates in the wettest quarter (wq_PER), indicating populations adapted to cooler and more humid highland environments. The corresponding localities are concentrated along the Ruwenzori and East African mountain chains. Cluster 3 lies in the lower left portion of the PCA space and is associated with higher values of the minimum temperature of the coldest month (bio6) and the mean temperature of the driest and coldest quarters (bio9, bio11). Spatially, Cluster 3 populations are distributed mainly in lowland and coastal regions, including parts of the Tanzanian Basin and the Indian Ocean coastal belt. Altogether, the identified clusters represent distinct ecological strategies of *G. pallidipes* populations across East Africa, shaped primarily by thermal tolerance limits and reproductive timing under seasonal climatic constraints (Fig. 7b).

### 3.5 Decision tree analysis results

Based on the classification decision tree constructed to identify the environmental variables that best discriminate between the ecological clusters of *G. pallidipes*, the most influential determinant was temperature annual range (bio7). Samples from regions with lower annual temperature variation were subsequently divided according to the mean temperature of the wettest quarter (bio8). Within this subset, samples characterized by relatively low wet-season temperatures were further distinguished by the Köppen Aridity Index (KAI). The lowest KAI values defined a small group assigned to Cluster 3, representing cooler and more humid environments. In contrast, moderately higher KAI values encompassed most samples (Cluster 2), corresponding to relatively warm, semi-humid conditions.

For the subset with higher wet-season temperatures, the key discriminator was the minimum temperature of the coldest month (bio6). Lower values identified a small group of Cluster 3 samples, indicative of cooler extremes even in otherwise warm regions, while higher values corresponded exclusively to Cluster 1, representing warm and thermally stable habitats.

Conversely, in regions with greater annual temperature variation, elevation emerged as the next most important factor. Lower-elevation environments were further partitioned by the precipitation of the wettest quarter (bio16). The lowest values marked a small set of Cluster 3 samples, while higher precipitation values corresponded to Cluster 1. In contrast, higher-elevation conditions were strongly associated with Cluster 3, suggesting that this group predominantly occupies cooler, highland regions characterized by broad annual temperature ranges.

**Fig. 8:**
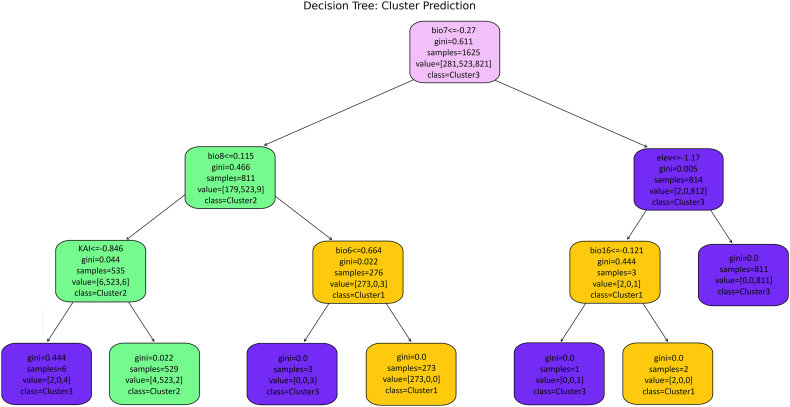
Decision tree analysis result.

Overall, the decision tree analysis revealed bio7, bio8, and KAI as the primary discriminators of ecological clusters, with bio6, elevation, and bio16 contributing smaller but meaningful effects. The results indicate that *G. pallidipes* populations are structured primarily along climatic gradients of temperature variability and humidity, with Cluster 1 linked to warm, stable environments; Cluster 2 associated with moderately humid, low-variability regions; and Cluster 3 occupying cooler, high-elevation or thermally variable habitats (Fig. 8)

### 3.6 Ecological space comparison of *Glossina pallidipes* and five mammal species

**Fig. 9:**
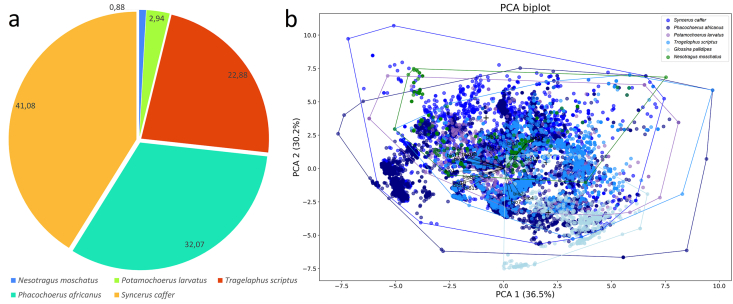
a: The occurrence of host African mammal species within the 0.5 x 0.5 arc-min presence grids of *Glossina pallidipes*. b: The PCA result of the evaluation of environmental conditions of *Glossina pallidipes*, *Nesotragus moschatus*, *Phacochoerus africanus*, *Potamochoerus larvatus*, *Syncerus caffer*, and *Tragelaphus scriptus*.

Within 0.5° × 0.5° grid cells where *G. pallidipes* is present, the occurrence frequencies of several important mammalian hosts were as follows: African buffalo: 41.08 %, common warthog: 32.07 %, bushbuck: 22.88 %, bushpig: 2.94 %, and suni antelope: 0.88 %. These results indicate that African buffalo and common warthog are the most frequently co-occurring hosts with *G. pallidipes* across the analysed spatial grid (Fig. 9a).

The principal component analysis (PCA) based on climatic, edaphic, and relief variables revealed clear ecological differentiation among the analysed mammal species and *G. pallidipes*. The first two principal components accounted for 66.7 % of the total environmental variance, with PC1 explaining 36.5 % and PC2 explaining 30.2 %. The distribution of the occurrence points in the biplot indicates partially overlapping but distinct ecological niches for the examined taxa. Along PC1, the primary separation is driven by variables associated with temperature gradients (e.g., bio1, bio4, and bio7) and precipitation seasonality (bio15), while PC2 reflects variation in moisture availability and soil properties (bio12, bio13, and bio16). Species such as African buffalo and common warthog are positioned toward the higher values of PC1, suggesting adaptation to warmer and more seasonal environments. In contrast, harnessed bushbuck and *G. pallidipes* occupy intermediate positions, reflecting tolerance to moderately humid and stable climatic conditions. Bushpig and suni show distinct clustering toward the upper range of PC2, associated with higher precipitation and denser vegetation cover, indicative of a preference for mesic habitats. The environmental vectors suggest that the climatic variables exert a stronger influence on species distribution than relief or edaphic factors, although the latter contribute to niche differentiation along minor axes. Convex hulls enclosing the points of each species show partial overlap, reflecting shared environmental preferences but also unique ecological optima. Overall, the PCA demonstrates that the studied taxa are distributed along a gradient from arid–open to humid–closed habitats, structured primarily by temperature and precipitation seasonality (Fig. 9b).

**Fig. 10:**
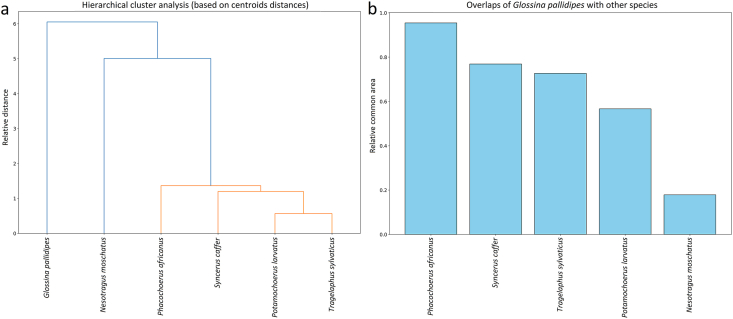
Hierarchical cluster analysis of the centroids of the species provided by PCA (a) and the ecological space overlaps of *Glossina pallidipes* with other species (b).

In the PCA-based analysis of environmental space, the centroid of *G. pallidipes* was found to be closest to that of suni, indicating a high similarity in ecological niche conditions (Fig. 10a). In contrast, the largest geographic overlap of convex hull areas occurred with common warthog, while overlap with suni was minimal (Fig. 10b). This apparent discrepancy arises from the difference between environmental similarity and spatial co-occurrence: the PCA centroids reflect similarity in environmental conditions, whereas geographic overlap reflects actual co-occurrence in space, which is influenced by the distribution and abundance of host species. Consequently, while *G. pallidipes* shares similar environmental preferences with suni, it is more likely to encounter common warthog in the field due to greater spatial overlap.

### 3.7 Phylogenetic results

A phylogenetic analysis based on cytochrome oxidase I (COI) gene sequences revealed a geographically structured pattern of genetic variation among *G. pallidipes* populations from eastern and southern Africa. The resulting cladogram displays well-supported clades corresponding broadly to regional origins, with several haplotypes exhibiting strong geographic clustering.

Most of the Ethiopian samples formed a highly distinct and deeply branching clade, separated from all other regional populations by relatively long branch lengths. This group includes multiple COI haplotypes (e.g., EthC10, EthC25, EthC64, and EthC88, EthC100) that clustered together with strong bootstrap support, suggesting a significant degree of regional endemism and genetic divergence in the northernmost population analysed.

In contrast, samples from Kenya, Tanzania, and the Serengeti region (South Kenya–North Tanzania) formed a more complex and interconnected assemblage. While some haplotypes grouped according to national boundaries, many exhibited close genetic affinities across the Kenya-Tanzania border. This likely reflects both gene flow and shared evolutionary history in the Serengeti ecosystem, where the tsetse fly populations are not constrained by political boundaries. These samples formed a large, relatively cohesive clade with several internal subclusters, including well-supported groups representing shared haplotypes (e.g., haplotype 2, haplotype 6, and haplotype 22), found in both Kenyan and Tanzanian individuals.

The Zimbabwean samples were positioned in a moderately distinct clade, branching between the Ethiopian lineage and the Serengeti-centered group. These samples (e.g., GpZC01, GpZC02) were closely related to a subset of Tanzanian haplotypes but retained unique genetic signatures, indicating partial isolation of the southern population and possibly reflecting historical separation or limited gene flow.

Additionally, the Gambian samples, although fewer in number, formed a small, well-supported clade (e.g., Gambia-2, Gambia-3) that was genetically distinct from all eastern and southern African populations. This finding supports the presence of localized genetic differentiation in western populations of *G. pallidipes*.

**Fig. 11:**
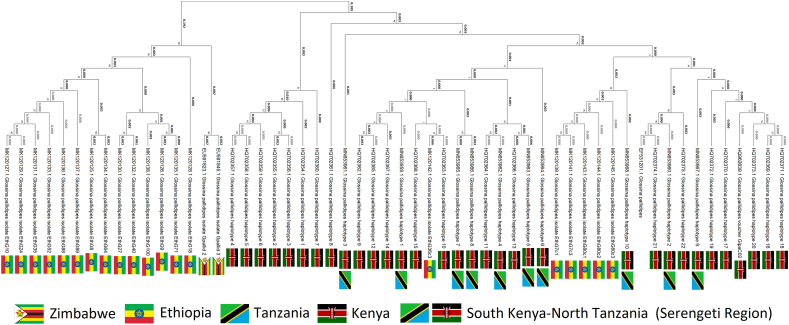
Geographic structure and phylogenetic relationships of *Glossina pallidipes* based on cytochrome oxidase I (COI) gene sequences.

Overall, the phylogenetic structure inferred from COI data demonstrates that *G. pallidipes* exhibits notable phylogeographic structuring, with distinct evolutionary lineages corresponding to Ethiopia, Zimbabwe, Gambia, and the Kenya–Tanzania–Serengeti region. The latter appears to be a genetic hotspot characterized by both haplotype diversity and inter-regional connectivity, consistent with ecological continuity in the savanna mosaic of East Africa (Fig. 11).

## 4 Discussion

This study provides a comprehensive ecological, climatic, and genetic characterization of *G. pallidipes*, revealing insights into the species’ spatial distribution, environmental associations, and population structure across eastern and southern Africa. By integrating kernel density estimation, climate classification, ensemble modeling, ecological clustering, host co-occurrence analysis, and phylogenetic data, both regional patterns and microhabitat variability were captured, with the analysis highlighting the complex ecological niche and adaptive responses of *G. pallidipes* to heterogeneous environments.

Kernel density analysis identified high-elevation regions—the Ethiopian Highlands, East African Highlands, and the Highveld—as primary hotspots for *G. pallidipes* occurrence. These results are consistent with previous studies (Cecchi et al., 2024; Rogers and Randolph, 1991), which emphasized the species’ preference for well-vegetated, humid areas at moderate altitudes. Such habitats provide optimal conditions for adult survival, larval development, and host availability, while supporting the presence of herbivorous game species (Matawa et al., 2016). A secondary hotspot was detected in the Masai Steppe, a region previously considered marginal for tsetse survival (Jannin, 1999; Cecchi and Mattioli, 2009), suggesting that local microclimatic or vegetative refugia can create suitable conditions even in semi-arid areas. Vegetation plays a key role in maintaining humidity and water balance, underlining the importance of high-resolution spatial data and ecological modelling for assessing vector habitat suitability (Kleynhans and Terblanche, 2009; Muyobela et al., 2023). Peripheral low-density presence across southern Uganda, western Ethiopia, and eastern Democratic Republic of Congo likely reflects marginal populations, under-sampling, or seasonal fluctuations (Results 3.1).

It can be concluded that *G. pallidipes* thrives in areas with sufficient vegetation for resting, availability of wild and domestic hosts, and relatively undisturbed habitats (Ndegwa et al., 2001). Low anthropogenic pressure in very low-density rural zones may offer refuges where tsetse populations can persist with minimal disruption. For example, in the region of the Akagera National Park, Rwanda, the occurrences of both *G. pallidipes* and *G. morsitans* were limited to the protected region and its narrow surrounding area (Gashururu et al., 2021). In addition, the importance of *Glossina* development factors also highlighted the crucial role of the driest season conditions on *G. pallidipes* occurrence.

Climatic analysis indicated that most occurrences were in tropical savanna climates (Aw, ∼41.5 %), with additional records in semi-arid (BSh, ∼20.6 %) and subtropical highland (Cwa/Cwb/Cfb, ∼26.2 %) regions. This aligns with previous findings showing that seasonal climate patterns influence feeding cycles, pupal development, and adult mortality (Terblanche et al., 2008). Populations in Af (tropical rainforest) and Am (monsoon) zones were less frequent (∼6.5 % and ∼1.9 %, respectively), indicating occasional populations near dense forest edges or riverine systems (Vale and Torr, 2005). *G. pallidipes* is less tolerant to desiccation than *G. morsitans centralis*, and its survival is strongly influenced by temperature and humidity regimes (Kleynhans and Terblanche, 2011; Terblanche et al., 2006). These findings suggest that thermal buffering and moisture availability are critical determinants of local population persistence, particularly in transitional zones between tropical and subtropical climates. As it was mentioned, only a small proportion of records fall within areas classified as Mediterranean-type (Csb) climates. These records are restricted to high-elevation regions of the Ethiopian Highlands, which exhibit cool, wet winters and dry summers consistent with the Csb classification, rather than the classical Mediterranean zones of South Africa's Western Cape (Van den Bossche et al., 2010), where tsetse is absent. In a climatological sense, this region largely coincides with the western side of the occurrence of the dry Afromontane Forest and grassland complex (compare the maps of Beck et al., 2018; Friis et al., 2022).

Human-modified landscapes influence tsetse distribution primarily through changes in host availability. Most sites (71 %) occurred in very low-density rural areas, with an additional 18 % in low-density regions, reflecting the species’ persistence where suitable mammalian hosts are present (Results 3.2.2; Fig. 4). Prior studies confirm that rural, minimally disturbed landscapes serve as refuges for tsetse, with wooded savannas, riverine systems, and agricultural mosaics providing critical blood sources (Auty et al., 2016; Ndegwa et al., 2001; Gashururu et al., 2021). The decline of occurrence in areas exceeding five people per km^2^ is likely driven by reductions in host density due to human activities rather than direct avoidance of humans (Vale et al., 1986; Van den Bossche et al., 2010). Low human presence combined with heterogeneous vegetation provides a mosaic of microhabitats conducive to sustaining tsetse populations year-round.

Ensemble modelling confirmed that temperature-related variables, particularly temperature seasonality (bio4), temperature annual range (bio7), and diurnal temperature range (bio2), consistently dominated predictive importance, followed by precipitation of the coldest quarter (bio19) and precipitation seasonality (bio15) (Results 3.3.1–3.3.2; Fig. 4, Fig. 5, Fig. 6; Tables 1–2). Developmental predictors, notably larviposition rates during the dry quarter (dp_PER), and mortality rates (dq_AFM, dq_PMR) were secondary but consistent determinants, emphasizing the interaction between thermal variability, humidity, and reproductive success (Pagabeleguem et al., 2016). Soil factors such as vertisols (soil_32) and fluvisols (soil_85) also contributed, likely reflecting moisture-retaining properties favourable for puparial survival (Gachoki et al., 2021). Accuracy and F1-score metrics for ensemble models demonstrated robust predictive performance, with Random Forest and Voting Ensemble methods achieving the highest reliability, underscoring the importance of combining climatic, edaphic, and development-related predictors for habitat suitability modelling.

Clustering analysis revealed three discrete ecological groups of *G. pallidipes*, structured mainly along altitudinal and thermal gradients rather than latitude (Rogers, 1988). These clusters reflect adaptive responses to regional climatic and topographic variability, particularly thermal regimes and moisture availability. Cluster 1 occurs in the Ethiopian Highlands and central East Africa, where moderate temperatures and stable, moist conditions support thermally buffered populations (Viste and Sorteberg, 2013). Cluster 2 occupies cooler, humid highlands, including the Ruwenzori and East African mountain chains, showing adaptations to extended pupal development during wet periods (Luhunga and Mutayoba, 2013). Cluster 3 is associated with warmer lowland and coastal regions, such as the Tanzanian Basin and Indian Ocean coast, where higher minimum and seasonal temperatures allow extended activity. These clusters illustrate three adaptive strategies shaped by thermal tolerance and reproductive timing under seasonally variable climates. It is well fit to the finding that *Glossina* flies acted out their strategy for survival against the background of seasonal changes (Rogers and Randolph, 1985). Field and laboratory studies on *G. pallidipes* indicate that survival and pupal development are highly temperature-dependent, with optimal reproduction occurring under thermally buffered conditions (Terblanche et al., 2008).Decision tree analysis further supported and corroborated these patterns, identifying the Köppen Aridity Index (KAI), elevation, bio4, bio7, bio19, and bio2 as key determinants. Cluster 1 was linked to warm, stable habitats; Cluster 2 to moderately humid, semi-arid highlands; Cluster 3 to cooler, high-elevation or thermally variable sites; and Cluster 4 to lowland, semi-arid areas with localized refugia (Nnko et al., 2021). These results illustrate environmental filtering along aridity and thermal gradients, shaping population differentiation across East Africa. Notably, aridity thresholds effectively separated populations in marginal zones, while elevation and seasonal temperature parameters accounted for fine-scale ecological partitioning, emphasizing the interplay between macro- and microclimatic constraints. The seasonal significance of microclimatic factors in *G. pallidipes* ecology has been recognized since the earliest studies (Vanderplank, 1948).

Phylogenetic analysis based on cytochrome oxidase I (COI) sequences demonstrated strong regional structuring. Similar, geographically and genetically distinct populations of this tsetse fly species were found, e.g., in Southern Kenya (Okeyo et al., 2018). Most *G. pallidipes* populations in Ethiopia formed a deeply divergent clade, suggesting long-term isolation and potential endemism. This pattern aligns with previous genetic studies (Krafsur, 2002; Solano et al., 2010), which identified the Ethiopian Highlands as a possible refugium (Leta et al., 2015), preserving unique genetic lineages due to geographic and ecological barriers. In contrast, populations from Kenya and Tanzania exhibited greater genetic connectivity, reflecting ecological continuity within the Serengeti–Mara ecosystem—a region characterized by extensive host migrations and continuous vegetation corridors. These landscape features are mirrored in the genetic structure of tsetse fly populations in the area (Solano et al., 2000). A similar, though less pronounced, genetic distinction between northwestern and southwestern populations (associated with the Serengeti ecosystem) is also supported by Bateta et al. (2020). Populations from Zimbabwe and The Gambia showed moderate to strong genetic differentiation, likely shaped by historical barriers to gene flow, such as fragmentation of habitat and regional climatic shifts during the Late Quaternary (Mairal et al., 2017). These findings point to a complex phylogeographic history, with multiple divergence events influenced by both biogeographic isolation and ecological adaptation.

Host association analysis based on geographical overlapping revealed that African buffalo (41.1 %) and common warthog (32.1 %) were the most frequently co-occurring mammals with *G. pallidipes*. This strong spatial association is consistent with feeding pattern studies, which have shown that *G. pallidipes* frequently feeds on African buffalo and common warthog, as confirmed by blood meal analyses (Muturi et al., 2011; Nyingilili et al., 2016; Ogolla et al., 2023). PCA of environmental space showed that *G. pallidipes* occupies an intermediate niche relative to other mammalian hosts, overlapping partially with *S. caffer* and *Ph. africanus*, but displaying the closest environmental similarity to suni. This distinction highlights that ecological similarity does not necessarily equate to spatial co-occurrence, with geographic overlap influenced by host abundance and distribution (Ogolla et al., 2023). Environmental axes, particularly temperature and precipitation seasonality, explained the majority of variance in niche differentiation, whereas soil and relief variables contributed to finer niche structuring, indicating that climatic gradients are primary drivers of habitat suitability.

Among the selected host species, African buffalo and bushpig show a strong ecological association with wetlands, riverine habitats, and floodplains (Cumming, 1975; Prins and Prins, 1996). E), whereas harnessed bushbuck is typically linked to riverine forest edges (Dankwa-Wiredu, 2000). In contrast, common warthog and suni primarily use water sources for drinking but generally inhabit drier savanna or thicket environments rather than wetlands (Treydte, 2004; Amin et al., 2015). These patterns closely mirror the already mentioned general habitat preferences of *G. pallidipes*. However, it should be added that feeding preferences vary regionally. For example, in the Serengeti, buffalo and elephant comprised 91.3 % of blood meals, whereas in Nguruman, buffalo alone accounted for 71.3 %, with no human blood detected (Nyingilili et al., 2016; Channumsin et al., 2021). In Shimba Hills, host diversity included domestic animals and occasional human blood, with temporal variation observed between years (Channumsin et al., 2021; Ogolla et al., 2023). These observations underscore the influence of local host availability and landscape heterogeneity on tsetse feeding ecology. Co-occurrence patterns suggest that *G. pallidipes* preferentially targets abundant wild ungulates, while human settlements and livestock density modulate feeding opportunities, further influencing survival and reproduction.

## 5 Conclusions

The ecological and genetic complexity underlying the distribution of *G. pallidipes* across eastern and southern Africa has been highlighted in this study. Distinct ecological clusters and phylogeographic patterns were identified through the integration of spatial modelling, climatic classification, and genetic analysis, we identified distinct ecological clusters and phylogeographic patterns that reflect regional environmental filtering and historical isolation. Environmental, climatic, and genetic evidence was jointly used to demonstrate that *G. pallidipes* populations are structured along multiple ecological axes, including temperature seasonality, precipitation patterns, aridity, and elevation. Thermal and moisture dynamics were found to interact with vegetation, soil type, and host distribution, shaping occurrence patterns in a way that reflects adaptive strategies to regional environmental variability. The strong association of *G. pallidipes* with tropical savanna climates, moderate elevations, and specific precipitation and temperature regimes was revealed emphasizing the need for local environmental contexts to be considered in surveillance and control efforts. Ensemble modelling and phylogenetic analyses confirmed that these patterns are ecologically meaningful and consistent with evolutionary divergence and connectivity, thereby demonstrating the value of integrative approaches for understanding vector ecology.

The detection of genetically distinct Ethiopian populations further indicates that vector control strategies should be adapted to regional ecological and evolutionary contexts rather than being implemented through uniform approaches. Collectively, these findings provide a framework through which the species’ response to future climate change—including potential shifts in distribution, host associations, and population dynamics—can be predicted. Ultimately, the successful management of *G. pallidipes* is likely to be achieved only through a One Health strategy in which ecological, climatic, and genetic information is integrated to identify refugia, predict range expansions, and optimize targeted, sustainable interventions against African trypanosomiasis (Dicko et al., 2014; Pagabeleguem et al., 2016; Gachoki, 2024).

## CRediT authorship contribution statement

**Attila J. Trájer:** Writing – review & editing, Writing – original draft, Visualization, Validation, Supervision, Software, Resources, Methodology, Investigation, Funding acquisition, Formal analysis, Data curation, Conceptualization. **Alex Kummer:** Writing – review & editing, Validation, Supervision, Software.

## Declaration of competing interest

The author declares that he has no known competing financial interests or personal relationships that could have appeared to influence the work reported in this paper.

## Figures and Tables

**figs1:**
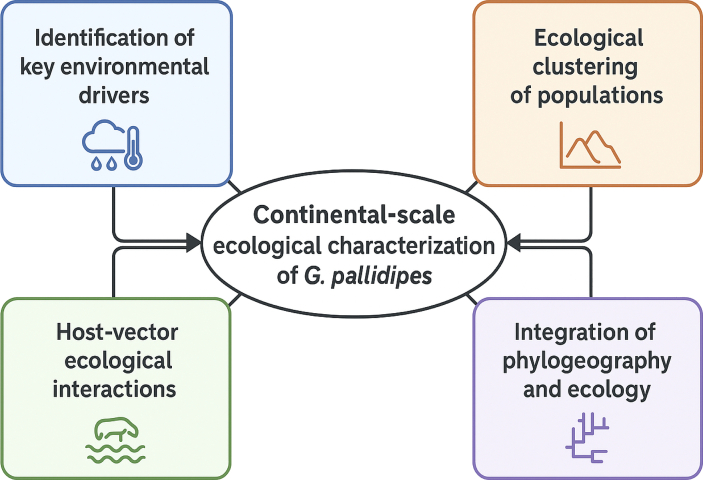


**figs2:**
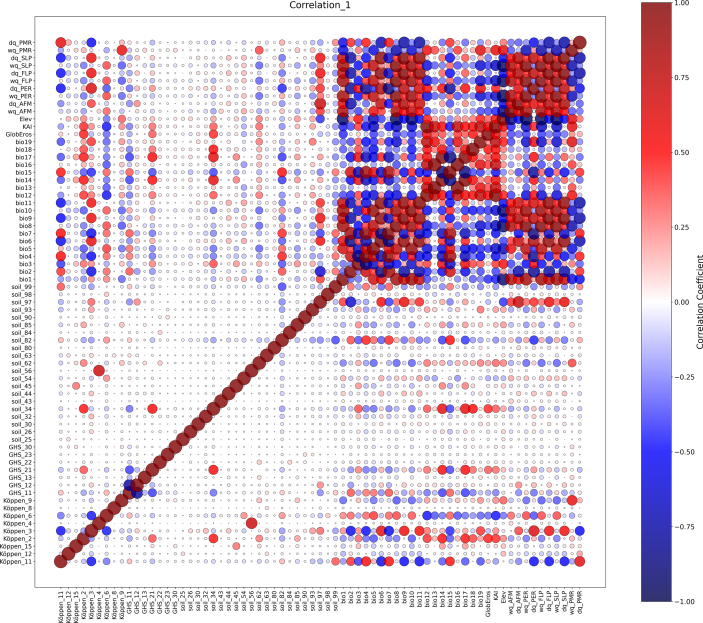


**figs3:**
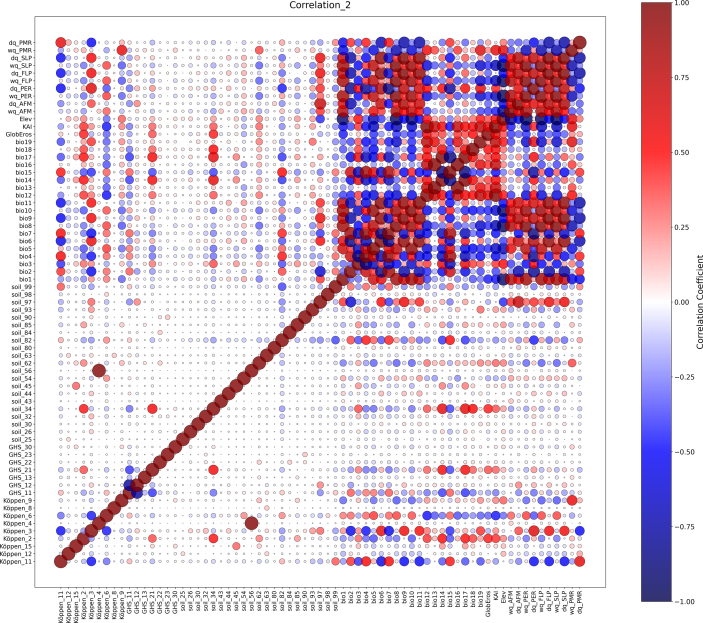


**figs4:**
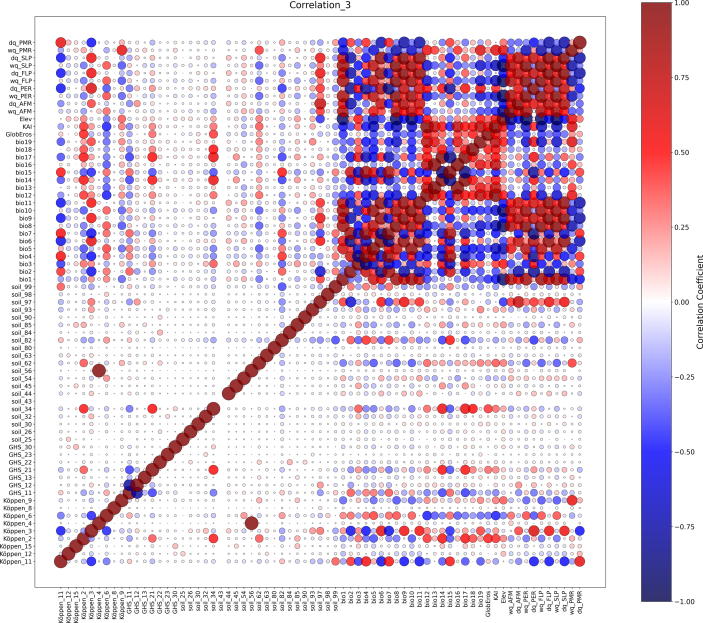


**figs5:**
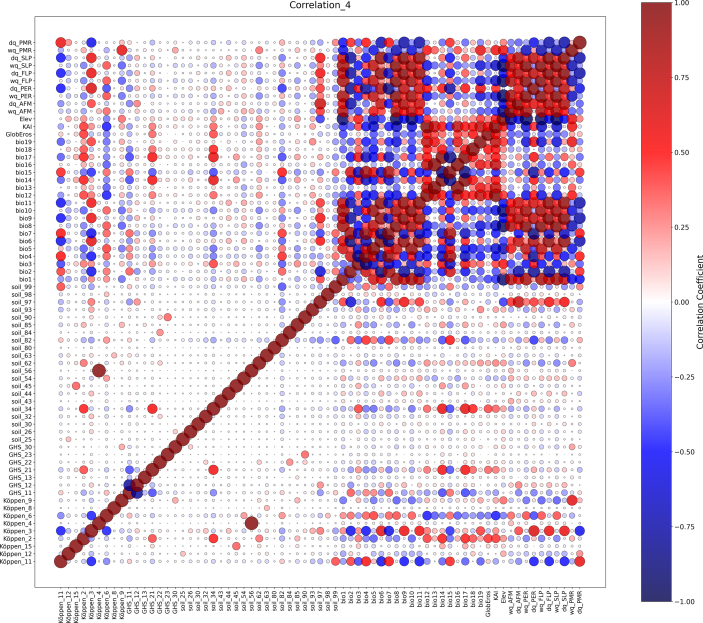


**figs6:**
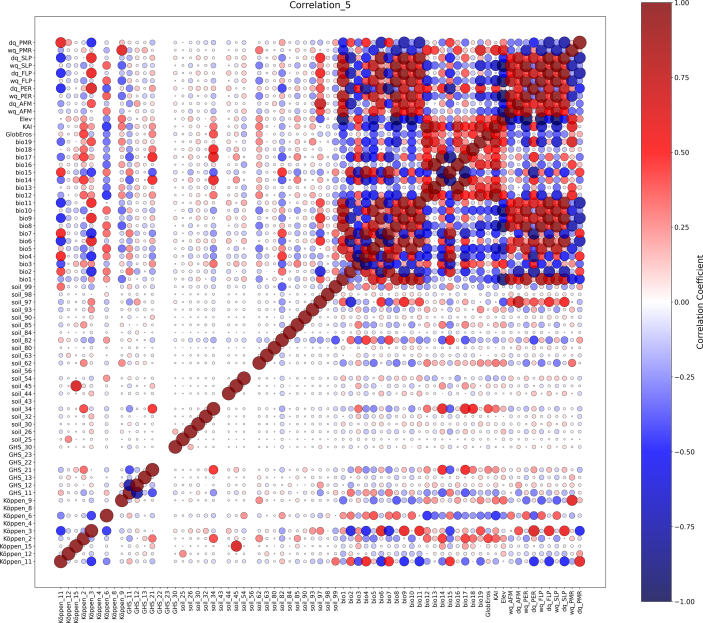


**figs7:**
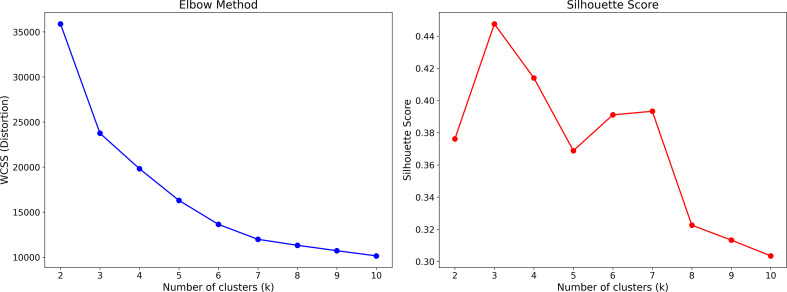


## Data Availability

Each piece of data used for the research can be found in the tables, supplementary materials, and referenced data sources.
