# Supplementary material for: Ecological zonation and phylogeographic structure of Glossina pallidipes (Diptera: Glossinidae) in eastern and southern Africa
Source: Int J Parasitol Parasites Wildl. 2025 Nov 22;28:101165. doi: 10.1016/j.ijppaw.2025.101165 (PMC12702227; doi:10.1016/j.ijppaw.2025.101165)
Supplement: Multimedia component 1 [file mmc1.docx]

**Supplementary Table 1**. Variables used in the analyses.

| **Abbreviation** | **Factor** | **Unit** | **Nature** | **Encoding** |
| --- | --- | --- | --- | --- |
| **Bioclimatic variables** | | | |  |
| Annual Mean Temperature | bio1 | °C | numerical | - |
| Mean Diurnal Range | bio2 | °C | numerical | - |
| Isothermality | bio3 | °C | numerical | - |
| Temperature Seasonality | bio4 | °C | numerical | - |
| Max Temperature of Warmest Month | bio5 | °C | numerical | - |
| Min Temperature of Coldest Month | bio6 | °C | numerical | - |
| Temperature Annual Range | bio7 | °C | numerical | - |
| Mean Temperature of Wettest Quarter | bio8 | °C | numerical | - |
| Mean Temperature of Driest Quarter | bio9 | °C | numerical | - |
| Mean Temperature of Warmest Quarter | bio10 | °C | numerical | - |
| Mean Temperature of Coldest Quarter | bio11 | °C | numerical | - |
| Annual Precipitation | bio12 | mm | numerical | - |
| Precipitation of Wettest Month | bio13 | mm | numerical | - |
| Precipitation of Driest Month | bio14 | mm | numerical | - |
| Precipitation Seasonality | bio15 | mm | numerical | - |
| Precipitation of Wettest Quarter | bio16 | mm | numerical | - |
| Precipitation of Driest Quarter | bio17 | mm | numerical | - |
| Precipitation of Warmest Quarter | bio18 | mm | numerical | - |
| Precipitation of Coldest Quarter | bio19 | mm | numerical | - |
| **FAO soil orders (suborders)** | | | | |
| soils_25 | Andisols, Ustands | - | categorical | one-hot |
| soils_26 | Andisols, Udands | - | categorical | one-hot |
| soils_30 | Oxisols, Aquox | - | categorical | one-hot |
| soils_32 | Oxisols, Ustox | - | categorical | one-hot |
| soils_34 | Oxisols, Udox | - | categorical | one-hot |
| soils_43 | Vertisols, Torrerts | - | categorical | one-hot |
| soils_44 | Vertisols, Usterts | - | categorical | one-hot |
| soils_45 | Vertisols, Uderts | - | categorical | one-hot |
| soils_54 | Aridisols, Argids | - | categorical | one-hot |
| soils_56 | Aridisols, Cambids | - | categorical | one-hot |
| soils_62 | Ultisols, Udults | - | categorical | one-hot |
| soils_63 | Ultisols, Ustults | - | categorical | one-hot |
| soils_80 | Alfisols, Aqualfs | - | categorical | one-hot |
| soils_82 | Alfisols, Ustalfs | - | categorical | one-hot |
| soils_84 | Alfisols, Udalfs | - | categorical | one-hot |
| soils_85 | Inceptisols, Udepts | - | categorical | one-hot |
| soils_90 | Inceptisols, Aquepts | - | categorical | one-hot |
| soils_93 | Inceptisols, Ustepts | - | categorical | one-hot |
| soils_97 | Entisols, Psamments |  | categorical | one-hot |
| soils_98 | Entisols, Fluvents | - | categorical | one-hot |
| soils_99 | Entisols, Orthents | - | categorical | one-hot |
| **Köppen-Geiger climatic classes** | | | | |
| Köppen_1 | Af; Tropical, rainforest | - | categorical | one-hot |
| Köppen_2 | Am; Tropical, monsoon | - | categorical | one-hot |
| Köppen_3 | Aw, Tropical, savannah | - | categorical | one-hot |
| Köppen_4 |  |  |  |  |
| Köppen_5 | BWk, Arid, desert, cold | - | categorical | one-hot |
| Köppen_6 | BSh, Arid, steppe, hot | - | categorical | one-hot |
| Köppen_7 | BSk, Arid, steppe, cold | - | categorical | one-hot |
| Köppen_8 | Csa, Temperate, dry summer, hot summer | - | categorical | one-hot |
| Köppen_9 | Csb, Temperate, dry summer, warm summer | - | categorical | one-hot |
| Köppen_10 | Csc, Temperate, dry summer, cold summer | - | categorical | one-hot |
| Köppen_11 | Cwa, Temperate, dry winter, hot summer | - | categorical | one-hot |
| Köppen_12 | Cwb, Temperate, dry winter, warm summer | - | categorical | one-hot |
| Köppen_13 | Cwc, Temperate, dry winter, cold summer | - | categorical | one-hot |
| Köppen_14 | Cfa, Temperate, no dry season, hot summer | - | categorical | one-hot |
| Köppen_15 | Cfb, Temperate, no dry season, warm summer | - | categorical | one-hot |
| Köppen_17 | Dsa, Cold, dry summer, hot summer | - | categorical | one-hot |
| Köppen_18 | Dsb, Cold, dry summer, warm summer | - | categorical | one-hot |
| Köppen_19 | Dsc, Cold, dry summer, cold summer | - | categorical | one-hot |
| **Topographical-related** | | | | |
| elev | elevation | m (a.s.l) | numerical | - |
| GlobEros | rainfall erosivity | . | numerical | - |
| **Global Human Settlement Layer Settlement Model classes** | | | | |
| GHSL_11 | Very low density rural | - | categorical | one-hot |
| GHSL_12 | Low density rural | - | categorical | one-hot |
| GHSL_13 | Rural cluster | - | categorical | one-hot |
| GHSL_21 | Suburban or peri-urban | - | categorical | one-hot |
| GHSL_22 | Semi-dense urban cluster | - | categorical | one-hot |
| GHSL_23 | Dense urban cluster | - | categorical | one-hot |
| GHSL_30 | Urban centre | - | categorical | one-hot |
| **Glossina development factors** | | | | |
| wq_AFM | Adult female mortality rate in the wettest quarter | day^-1^ | numerical | - |
| dq_AFM | Adult female mortality rate in the driest quarter | day^-1^ | numerical | - |
| wq_FLP | Larviposition rate — first larviposition in the wettest quarter | day^-1^ | numerical | - |
| dq_FLP | Larviposition rate — first larviposition in the driest quarter | day^-1^ | numerical | - |
| wq_PER | Pupal emergence rate in the wettest quarter | day^-1^ | numerical | - |
| dq_PER | Pupal emergence rate in the wettest quarter | day^-1^ | numerical | - |
| wq_PMR | Pupal mortality  rate in the wettest quarter | day^-1^ | numerical | - |
| dq_PMR | Pupal mortality  rate in the wettest quarter | day^-1^ | numerical | - |
| wq_SLP | Larviposition rate — subsequent larviposition in the wettest quarter | day^-1^ | numerical | - |
| dq_SLP | Larviposition rate — subsequent larviposition in the driest quarter | day^-1^ | numerical | - |
