# Supplementary material for: Ecological zonation and phylogeographic structure of Glossina pallidipes (Diptera: Glossinidae) in eastern and southern Africa
Source: Int J Parasitol Parasites Wildl. 2025 Nov 22;28:101165. doi: 10.1016/j.ijppaw.2025.101165 (PMC12702227; doi:10.1016/j.ijppaw.2025.101165)
Supplement: Multimedia component 2 [file mmc2.docx]

**Supplementary Table S2**. The accuracy and F1-score results of ensemble methods.

| **Ensemble method** | **Accuracy** | **F1-score** |
| --- | --- | --- |
| Random Forest | 0.826 | 0.826 |
| XGBoost | 0.807 | 0.807 |
| Gradient Boosting | 0.783 | 0.782 |
| Extra Trees | 0.807 | 0.806 |
| Voting Ensemble | 0.825 | 0.824 |
