# Supplementary material for: Ecological zonation and phylogeographic structure of Glossina pallidipes (Diptera: Glossinidae) in eastern and southern Africa
Source: Int J Parasitol Parasites Wildl. 2025 Nov 22;28:101165. doi: 10.1016/j.ijppaw.2025.101165 (PMC12702227; doi:10.1016/j.ijppaw.2025.101165)
Supplement: Multimedia component 3 [file mmc3.docx]

**Supplementary Table S3**. Accuracy and F1-score of ensemble models across climatic, developmental, Köppen, and edaphic factor groups.

| **Bioclimatic factors and Köppen aridity index** | | |
| --- | --- | --- |
| **Ensemble method** | **Accuracy** | **F1-score** |
| Random Forest | 0.807 | 0.806 |
| XGBoost | 0.802 | 0.802 |
| Gradient Boosting | 0.764 | 0.762 |
| Extra Trees | 0.807 | 0.807 |
| Voting Ensemble | 0.810 | 0.810 |
| **Development factors** | | |
| **Ensemble method** | **Accuracy** | **F1-score** |
| Random Forest | 0.686 | 0.687 |
| XGBoost | 0.703 | 0.702 |
| Gradient Boosting | 0.699 | 0.697 |
| Extra Trees | 0.669 | 0.670 |
| Voting Ensemble | 0.701 | 0.701 |
| **Köppen factors** | | |
| **Ensemble method** | **Accuracy** | **F1-score** |
| Random Forest | 0.548 | 0.549 |
| XGBoost | 0.543 | 0.530 |
| Gradient Boosting | 0.543 | 0.530 |
| Extra Trees | 0.543 | 0.530 |
| Voting Ensemble | 0.543 | 0.530 |
| **Edaphic factors** | | |
| **Ensemble method** | **Accuracy** | **F1-score** |
| Random Forest | 0.588 | 0.581 |
| XGBoost | 0.588 | 0.581 |
| Gradient Boosting | 0.579 | 0.565 |
| Extra Trees | 0.588 | 0.581 |
| Voting Ensemble | 0.588 | 0.581 |
